# Alkaline Hydrolysis of Waste Acrylic Fibers Using the Micro-Water Method and Its Application in Drilling Fluid Gel Systems

**DOI:** 10.3390/gels9120974

**Published:** 2023-12-13

**Authors:** Wenjun Long, Zhongjin Wei, Fengshan Zhou, Shaohua Li, Kang Yin, Yu Zhao, Siting Yu, Hang Qi

**Affiliations:** Beijing Key Laboratory of Materials Utilization of Nonmetallic Minerals and Solid Wastes, National Laboratory of Mineral Materials, School of Materials Science and Technology, China University of Geosciences (Beijing), No. 29 Xueyuan Road, Haidian District, Beijing 100083, China; longwj@cugb.edu.cn (W.L.); 3003190032@cugb.edu.cn (Z.W.); 2103210080@email.cugb.edu.cn (S.L.); 2103220083@email.cugb.edu.cn (K.Y.); 2103210079@email.edu.cn (Y.Z.); 2103210078@email.cugb.edu.cn (S.Y.); 2003220043@email.cugb.edu.cn (H.Q.)

**Keywords:** waste acrylic fiber, hydroplastization, micro-water method, alkaline hydrolysis, filtrate reducer

## Abstract

Filtrate reducer is a drilling fluid additive that can effectively control the filtration loss of drilling fluid to ensure the safe and efficient exploitation of oilfields. It is the most widely used treatment agent in oilfields. Due to its moderate conditions and controllable procedure, alkaline hydrolysis of high-purity waste polyacrylonitrile has been utilized for decades to produce filtrate reducer on a large scale in oilfields. However, the issues of long hydrolysis time, high viscosity of semi-finished products, high drying cost, and tail gas pollution have constrained the development of the industry. In this study, low-purity waste acrylic fiber was first separated and purified using high-temperature hydroplastization, and the hydrolyzed product was obtained using alkaline hydrolysis with the micro-water method, which was called MW−HPAN. The hydrolysis reaction was characterized using X-ray diffraction, scanning electron microscopy, infrared spectroscopy, and thermogravimetric analysis, and the elemental analysis showed a hydrolysis degree of 73.21%. The experimental results showed that after aging at 180 °C for 16 h, the filtration volume of the freshwater base slurry with 0.30% dosage and 4% brine base slurry with 1.20% dosage was 12.7 mL and 18.5 mL, respectively. The microstructure and particle size analysis of the drilling fluid gel system showed that MW−HPAN could prevent the agglomeration of clay and maintain a reasonable particle size distribution even under the combined deteriorating effect of high temperature and inorganic cations, thus forming a dense filter cake and achieving a low filtrate volume of the drilling fluid gel system. Compared with similar commercially available products, MW−HPAN has better resistance to temperature and salt in drilling fluid gel systems, and the novel preparation method is promising to be extended to practical production.

## 1. Introduction

The trend toward fast fashion and increased fiber consumption in the apparel industry has led to more frequent iterations of apparel products and shorter life cycles for textiles, generating large amounts of textile waste [1]. Textile waste is mainly generated during the textile production process, as well as during the use and disposal by consumers, and has a complex composition, being a mixture of different fibers, which is difficult to degrade in the natural environment, and coarse landfilling and incineration will pollute the atmosphere [2]. At present, the global annual production of used textiles is more than 40 million tons, and China’s annual production is more than 26 million tons, accounting for about 6% of the total municipal solid waste [3,4]. However, due to the lack of a recycling system for used textiles and public awareness of environmental protection, as well as the relative backwardness of China’s recycling technology, the overall recycling rate of used textiles in China is less than 10% [5], which is far below the level of the most developed countries [6].

Acrylic fiber is one of the most widely used textile raw materials due to its softness, thermal stability, and other advantages. China’s acrylic fiber production ranks fourth among synthetic fibers, accounting for about 0.79%, with an average annual production of about 650,000 tons in the past ten years, in addition to more than 100,000 tons of imported acrylic fiber products every year (data from the website of the National Bureau of Statistics of China); thereby, a considerable amount of waste acrylic fiber is produced every year. The main chemical composition of waste acrylic fibers is polyacrylonitrile, where the polyacrylonitrile content varies from different sources. Waste acrylic fibers are difficult to degrade and cannot be naturally degraded or thermoformed. The incineration of these fibers produces toxic gases such as HCN and NH_3_, which pollute the environment. Under the effect of acid, alkali, heat, and pressure, the hydrophobic cyano functional groups on the polyacrylonitrile molecular chain with high chemical reactivity can be hydrolyzed to generate amide and carboxyl groups to obtain functional polymer materials [7].

**Figure 1:**
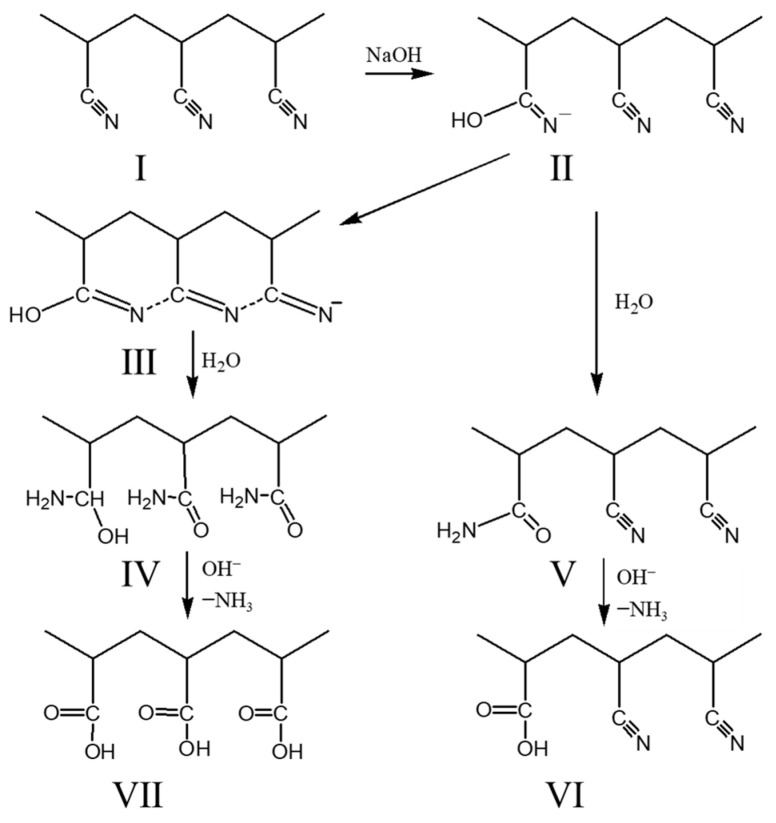
Mechanism of alkaline hydrolysis of polyacrylonitrile [14].

Hydrolyzed polyacrylonitrile products (HPAN) have been used as oilfield additives for several decades, mainly as a filtrate reducer for drilling fluid gel systems. The raw material for the preparation was changed from pure polyacrylonitrile to waste acrylic fibers to reduce the cost, and alkaline hydrolyzed polyacrylonitrile was used for large-scale industrial production due to the advantages of moderate conditions and controllable process. Over the past sixty years, scientists have conducted a number of studies on the mechanism underlying the alkaline hydrolysis reaction of polyacrylonitrile [8,9,10,11,12,13]. It is now generally accepted that the alkaline hydrolysis of polyacrylonitrile produces six-membered ring reaction intermediates (Figure 1) and that the properties of the hydrolysis products are closely related to the hydrolysis degree.

Solvents for the reaction of water-soluble polymers have a great effect on the efficiency and stability of the reaction. Take the starch modification reaction as an example, many research studies have been carried out in the field of the chemical modification of starch. Most chemical modifications of starch, such as esterification and carboxymethylation, are wet modifications, i.e., carried out in sufficient aqueous or organic solvents, including ethanol [15,16], N,N-dimethylformamide [17], pyridine [18], etc. The aqueous solution reaction system has the disadvantages of poor reaction efficiency, unstable product quality, and increasing side reactions. The organic solvent method can improve the reaction efficiency, but the cost increases and it is toxic and easily causes environmental pollution [19]. Scientists have developed dry and semi-dry starch modification methods on this basis [20,21,22]. Zhu et al. [23] investigated the effect of different starch moistures on solvent-free esterification reactions. At low water contents, the esterification reaction is difficult due to strong hydrogen bonding, whereas at too high moisture, the degree of substitution and the efficiency of the reaction decrease due to side reactions and dilution. Karic et al. [24] modified starch without solvent in the moist/semi-moist state using betaine hydrochloride (BHC) as a cationic reagent, resulting in starch derivatives with sufficiently high cationicity. Compared with aqueous and organic solvent methods, solvent-free modification keeps starch in a solid state during processing, resulting in a significantly different contact interface with chemical reagents. Due to the low solvent content, the occurrence of side reactions is suppressed to the maximum extent, and the microenvironment of the reaction system is made different from that of liquid-phase reactions, resulting in a localized high concentration at the reaction site, which improves the efficiency of the reaction. In addition, the solvent-free reaction is also an energy-saving and environmentally friendly process, and it is necessary to extend it to other chemical modification methods for water-soluble polymer materials [25].

Due to the agglomeration and hydrophobicity of polyacrylonitrile fibers, the hydrolysis reaction is an inhomogeneous reaction in the initial stage, resulting in a slow reaction speed, and the high-water retention of hydrolyzed semi-finished products makes them difficult to dry. In addition, the reaction process continues to emit irritating ammonia gas. The development of polyacrylonitrile hydrolysis technology that reduces environmental pollution, improves production efficiency, and lowers production costs has become essential. Thus, there is a need to improve the hydrolysis process to meet the requirements of being an environmentally friendly and low-cost production process.

In this study, polyacrylonitrile was hydrolyzed using a novel micro-water method process, and the effects of different reaction conditions on the filter loss reduction properties of the samples were investigated to determine the optimum hydrolysis reaction conditions. The occurrence of the hydrolysis reaction was characterized using analytical techniques such as FT-IR and elemental analysis. The filtration loss performance of drilling fluid gel systems with samples after aging at different temperatures was investigated by testing filtration loss volume, and the morphology of the filter cake, the rheological property, and particle size distribution of the drilling fluid gel system were analyzed. Compared with commercially available conventional HPAN products, the samples show better temperature and salt resistance. This research can improve the utilization and alkaline hydrolysis rate of waste acrylic fibers. Furthermore, the hydrolysis reaction of polyacrylonitrile with the micro-water method is simple in operation, and it can avoid the problems of high energy consumption and high cost, which is a more environmentally friendly process than the conventional process. This novel micro-water hydrolysis process is expected to replace the conventional process and be scaled up to practical production.

## 2. Results and Discussion

### 2.1. Structure Characterization of MW−HPAN

The structures of the used acrylic powder (LN-PAN) and MW−HPAN were tested, analyzed, and compared using an organic elemental analyzer, X-ray diffractometer, scanning electron microscope, Fourier transform infrared spectrometer, and thermogravimetry. MW−HPAN samples used for the tests were prepared under optimum reaction conditions: m_PAN_:m_NaOH_:m_H2O_ = 1:0.5:0.6, the reaction temperature was 160 °C, and the reaction time was 1 h.

#### 2.1.1. Elemental Analysis

The organic elemental content of the samples before and after hydrolysis was determined using an organic elemental analyzer. According to the results shown in Table 1, after the hydrolysis reaction took place, there was a very significant decrease in the nitrogen content of the sample and a large increase in the C/N ratio, corresponding to the ammonia emitted by the cyano hydrolysis process. In addition, there was a large increase in the oxygen content and, therefore, a decrease in the C/O ratio, corresponding to the carboxyl groups produced during the hydrolysis process [26]. Based on the results of the elemental analysis and the conservation of the mass of carbon atoms, it was calculated that the mass of MW−HPAN after the reaction was 33.4 g. Therefore, 0.2221 mol NH_3_ was released during the hydrolysis of polyacrylonitrile, which corresponds to the molar amount of carboxyl groups produced. If all the cyano groups were hydrolyzed to carboxyl groups, theoretically, 0.3034 mol of carboxyl groups would be produced, resulting in 73.21% hydrolysis of MW−HPAN [27]. This suggests that a large proportion of the cyano groups are hydrolyzed to carboxyl groups, which is consistent with the subsequent FTIR spectrogram.

#### 2.1.2. XRD Analysis

**Figure 2:**
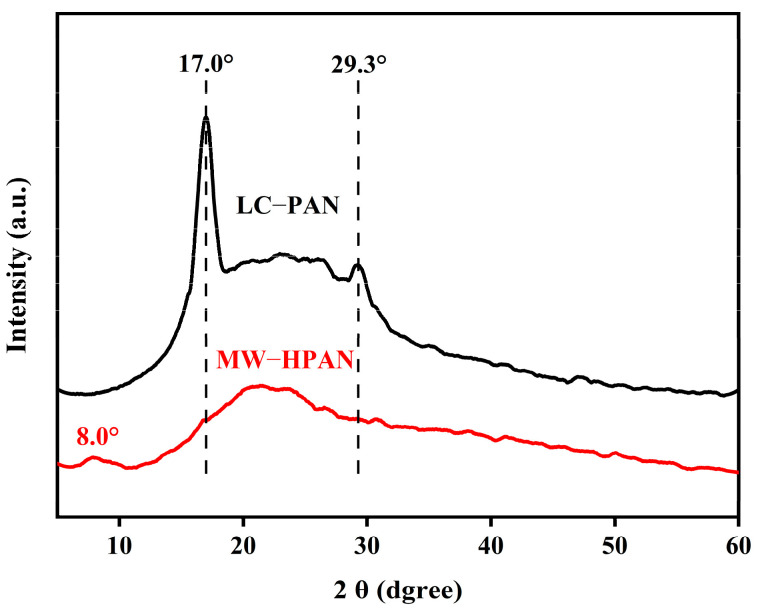
X-ray diffraction spectrograms of LC−PAN and MW−HPAN.

The changes in the crystallinity of the polyacrylonitrile powder before and after the hydrolysis reaction were determined using an X-ray diffractometer, and the results are shown in Figure 2. The XRD diffraction pattern of the original polyacrylonitrile powder shows a strong diffraction peak at 2θ = 17.0° and a weaker diffraction peak at 2θ = 29.3°, corresponding to the (100) and (101) plane, respectively [28]. Between the two diffraction peaks, there is a large and diffuse amorphous diffraction peak at about 2θ = 25.0°, which is the characteristic diffraction peak of polyacrylonitrile, corresponding to the coexistence of relatively ordered quasi-crystalline regions and disordered amorphous regions [29]. After hydrolysis, the XRD diffraction peaks of MW−HPAN at 2θ = 17.0° and 29.3° disappeared completely, indicating that the crystallinity of MW−HPAN was reduced. A new characteristic peak appeared at around 2θ = 8.0°, corresponding to the appearance of the amide peak from the hydrolysis of PAN [29]. During the hydrolysis reaction, part of the cyano group reacted with sodium hydroxide, resulting in the destruction of the relatively regular structure of the polyacrylonitrile molecule and leading to a reduction in crystallinity, which was conducive to the improvement of hydrophilicity.

#### 2.1.3. SEM Characterization

**Figure 3:**
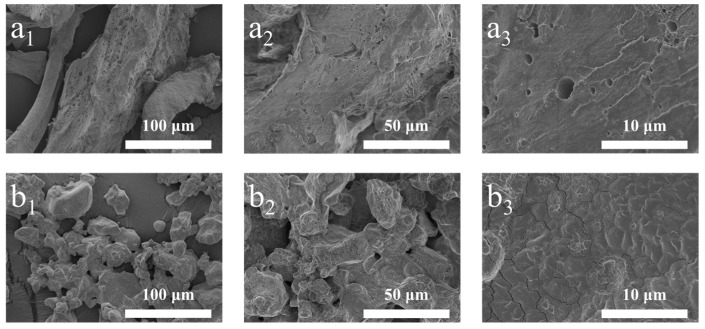
Scanning electron microscope images of LC−PAN and MW−HPAN. (**a_1_**) LC−PAN × 500, (**a_2_**) LC−PAN × 1.0 k, (**a_3_**) LC−PAN × 5.0 k, (**b_1_**) MW−HPAN × 500, (**b_2_**) MW−HPAN × 1.0 k, and (**b_3_**) MW−HPAN × 5.0 k.

The morphology of the polyacrylonitrile powder before and after the hydrolysis reaction is shown in Figure 3. Before the reaction, the polyacrylonitrile powder still had a fibrous structure with a relatively smooth surface and a small number of micropores (Figure 3a_1_–a_3_). After the reaction, the MW−HPAN powder was irregularly shaped, the particle size was reduced, the surface flatness was reduced and became rougher, and, at the same time, many cracks were formed (Figure 3b_1_–b_3_). The change in powder morphology confirms the decrease in crystallinity and the occurrence of the hydrolysis reaction.

#### 2.1.4. FT-IR Characterization

**Figure 4:**
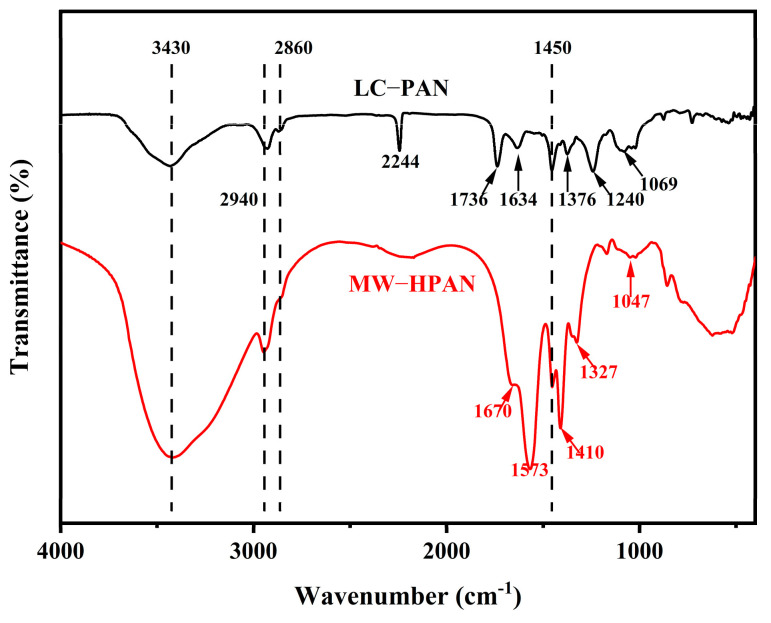
FT-IR spectrograms of LC−PAN and MW−HPAN.

Figure 4 shows the obvious changes in the infrared spectra of LC−PAN and MW−HPAN. The peaks at 2940 cm^−1^ and 2860 cm^−1^ on the spectra correspond to the asymmetric and symmetric stretching vibrations of the methylene group, respectively, and the peak at 1450 cm^−1^ is assigned to the metastable vibration of the methylene group [30]. A band at 2244 cm^−1^ is observed in the spectra of LC−PAN, which is the characteristic stretching vibration peak of −CN. The peak appears at 1736 cm^−1^, and 1634 cm^−1^ is assigned to the stretching vibration of carbonyl and -C=C, which is derived from copolymer monomer, and the peaks at 1376 cm^−1^ correspond to the bending vibration of the methylene group [31]. Compared with LC−PAN, the broader peak at 3430 cm^−1^ in the MW−HPAN spectrum corresponds to the N-H stretching vibration of the amide group generated by the hydrolysis reaction, and the peak at 2244 cm^−1^ almost completely disappeared, indicating the consumption of the cyano group [32,33]. The peak at 1067 cm^−1^ is the stretching vibration peak of the amide group, and the peaks at 1573 cm^−1^ and 1410 cm^−1^ are typical carboxylate vibration peaks corresponding to antisymmetric and symmetric stretching vibrations, respectively. The widening and intensification of the peak at 1047 cm^−1^ is due to the generation of the carboxylic acid group [34,35]. The changes in the infrared spectra indicate that the alkaline hydrolysis process consumes the ester group and most of the cyano group on the molecular chain of polyacrylonitrile and generates an amide group and a sodium carboxylate group.

#### 2.1.5. Thermogravimetric Analysis

**Figure 5:**
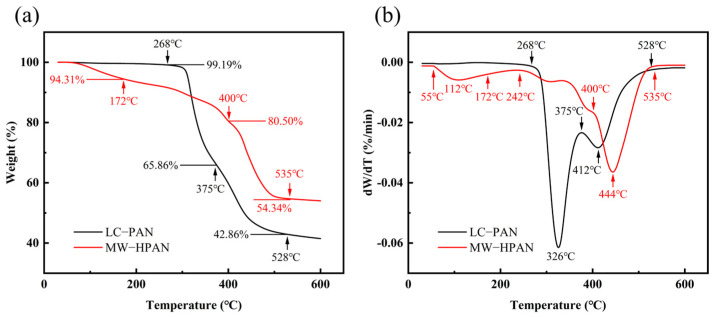
The thermal properties of LC−PAN and MW−HPAN. (**a**) Thermogravimetric (TG) analysis curves of LC−PAN and MW−HPAN (**b**) Differential thermogravimetric (DTG) curves of LC−PAN and MW−HPAN.

The thermal stability of LC−PAN and MW−HPAN was compared using thermogravimetric analysis. The TGA and DTG curves are displayed in Figure 5. The thermal decomposition process of both LC−PAN and MW−HPAN can be divided into three stages. The first mass loss stage of LC−PAN occurs before 268 °C and is attributed to the escape of HCN and NH_3_, which is produced by the splitting of side and end groups not involved in the cyclization reaction between adjacent cyanide groups. This stage transforms PAN from a linear into a trapezoidal structure, losing only 0.81% of its mass [36]. The first stage of MW−HPAN occurs before 172 °C, resulting in a mass loss of about 5.69%, which corresponds to the evaporation of bound water. The second stage of PAN occurs at 268 °C~375 °C and is due to the decomposition of the cyclic structure, which releases a large amount of hydrogen and methane, reaching the maximum rate of thermal decomposition at 326 °C, and the mass loss of the substance is 33.33% [37,38]. The second stage of MW−HPAN occurs at 172 °C~400°C, during which the cyclization and thermal decomposition of anhydride take place. MW−HPAN forms a more stable trapezoidal structure than the polyacrylonitrile molecule due to the additional ionic mechanism, resulting in a mass loss of 13.81%, which is lower than polyacrylonitrile [29]. The third stage of mass loss of LC−PAN and MW−HPAN occurs at 375 °C~528 °C and 400 °C~535 °C respectively, corresponding to the further oxidative decomposition of the residue [39]. The residual carbon amount of MW−HPAN at 600 °C is higher than that of LC−PAN, and the thermal decomposition behavior of MW−HPAN indicates its resistance to high temperatures.

#### 2.1.6. Particle Size Analysis

**Figure 6:**
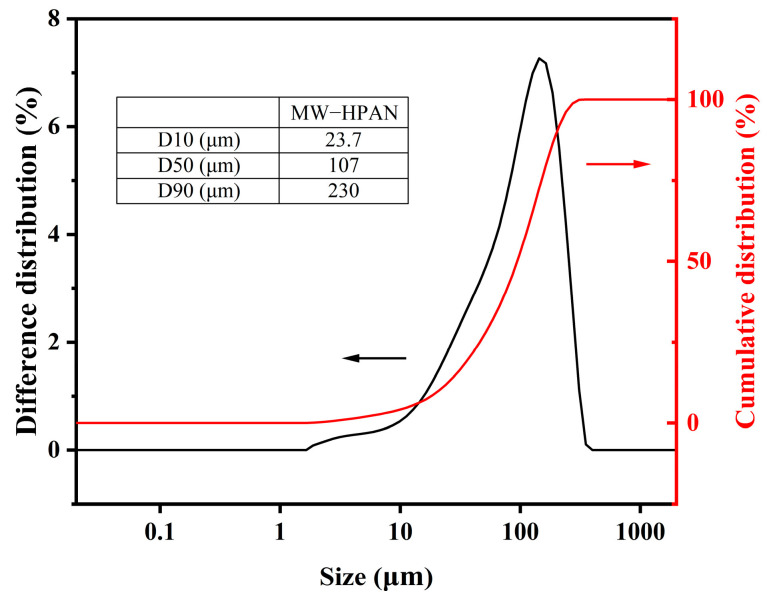
Particle size distribution of MW−HPAN.

The particle size distribution of the sample was measured with a laser particle sizer. The particle size of a polymer affects its dissolution rate in the drilling fluid: the larger the particle size, the slower the dissolution rate. The particle size distribution of the sample is shown in Figure 6. Overall, 50% of the samples have a particle size of less than 107 μm, and 90% of the particle sizes are less than 230 μm, indicating that particle sizes are small and can dissolve quickly, which is consistent with the experimental phenomenon.

### 2.2. Optimization of the Hydrolysis Parameters

The experimental parameters were optimized by testing the filtration loss performance of the samples in freshwater and a 4% brine base slurry after being cured at room temperature for 16 h. The dosage of freshwater and 4% brine base slurry were 0.3% and 1.2%, respectively.

#### 2.2.1. Effect of Water Addition on the Filtrate Reduction Property

**Figure 7:**
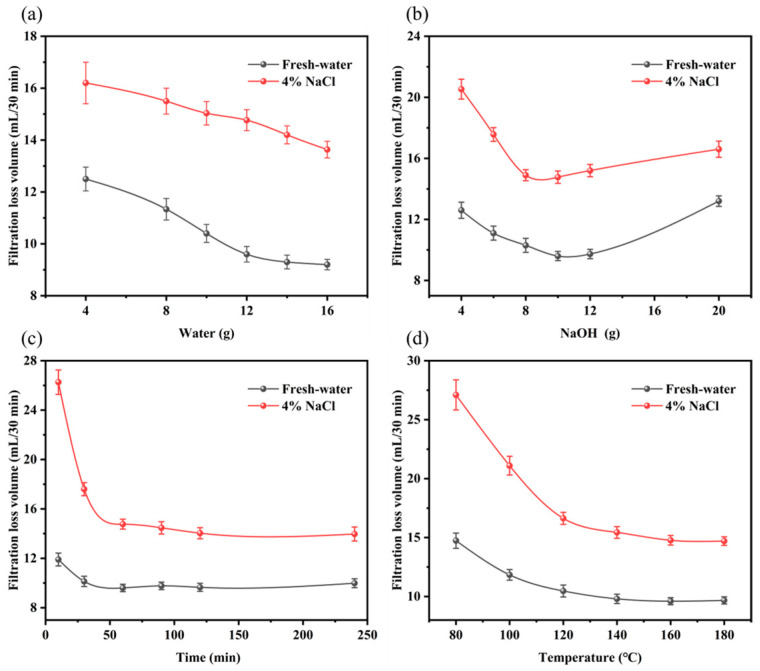
The room temperature filter loss volume of different experimental variable samples in the freshwater and 4% brine base slurry: (**a**) water addition, (**b**) NaOH addition, (**c**) reaction time, and (**d**) reaction temperature.

PAN was hydrolyzed with water addition dosages of 4 g, 8 g, 10 g, 12 g, 14 g, and 16 g, respectively, and the remaining experimental parameters remained unchanged. The results are shown in Figure 7a. The filter loss volume of the sample was reduced with the increase in water addition, indicating that the hydrolysis degree of the sample increased and the water solubility was improved, which suggests that the increase in water addition was beneficial to improving the filtration loss reduction performance of the sample. When the amount of water was 12 g, the filter loss volume of the sample in the fresh water and 4% brine base slurry was 14.7 mL and 9.6 mL, respectively, which could meet the actual requirements, and the sample could be pulverized without subsequent drying. When the water addition exceeded 12 g, the increase in water addition did not further improve the loss reduction performance of the sample, which would increase the cost of drying. Thus, 12 g was selected as the optimum water addition.

#### 2.2.2. Effect of NaOH Addition on the Filtrate Reduction Property

PAN was hydrolyzed under the conditions of sodium hydroxide dosages of 4 g, 6 g, 8 g, 10 g, 12 g, and 20 g, respectively, and the remaining variables were kept constant. When the sodium hydroxide addition was less than 10 g, the filtration loss volume decreased with the increase in sodium hydroxide dosage, indicating the hydrolysis degree of PAN and the water solubility of the sample increased. When the amount of sodium hydroxide was higher than 10 g, excessive caustic soda caused the degradation of PAN and the decrease in molecular weight of the sample increased the filtration loss. Therefore, the optimum amount of sodium hydroxide was determined to be 10 g (Figure 7b).

#### 2.2.3. Effect of Reaction Time on the Filtrate Reduction Property

PAN was hydrolyzed under the conditions of a reaction time of 10 min, 30 min, 60 min, 90 min, 120 min, and 240 min, respectively, and the other experimental parameters remained unchanged. As demonstrated in Figure 7c, when the reaction time was lower than 60 min, a significant reduction was shown in the filtration loss of the sample with the increase in the hydrolysis reaction time. This indicated that the hydrolysis degree of PAN increased, and the filtration loss reduction performance of the sample was improved. After the reaction time exceeded 60 min, the filtration loss of the drilling fluid gel system basically had no obvious change, which indicated that the increase in the reaction time at this time did not improve the hydrolysis degree of PAN, and the increase in the reaction time would increase the production cost. Therefore, the optimal reaction time was determined to be 60 min.

#### 2.2.4. Effect of Temperature on the Filtrate Reduction Property

PAN was hydrolyzed under the conditions of reaction temperatures of 80 °C, 100 °C, 120 °C, 140 °C, 160 °C, and 180 °C, respectively, and the other parameters remained unchanged. As shown in Figure 7d, when the reaction temperature was lower than 100 °C, most of the water molecules in the system existed in liquid form, the reaction was not uniform, and the reaction speed was slow, leading to a low hydrolysis degree and a high filtration loss volume (higher residues of acrylic powder were observed in the product). As the temperature rose, the water molecules were transformed into a gaseous state, which was conducive to contact with acrylic powder and improved the uniformity of the reaction, thus increasing the reaction speed and hydrolysis degree and improving the filtration loss reduction performance of MW−HPAN. When the reaction temperature was higher than 160 °C, there was no significant difference in the properties of the products, and the optimum reaction temperature was determined to be 160 °C, according to the experimental results.

### 2.3. Evaluation of the Filtration Control Property of MW−HPAN

The samples were prepared under optimal experimental conditions in order to comprehensively evaluate the filtration loss performance and action mechanism, rheological properties, filtration reduction performance, particle size distribution, and morphology of the filter cake in the freshwater base slurry and the 4% NaCl brine base slurry before and after aging at 180 °C for 16 h were analyzed.

#### 2.3.1. Rheological Properties of Drilling Fluid Gel Systems

A drilling fluid gel system is a kind of pseudoplastic fluid. The Bingham plastic model and the Herschel–Bulkely model were used to simulate and evaluate the multivariate fluid properties of drilling fluid gel systems scientifically [40].

Bingham plastic model:
  (1) $$\tau =\tau_{0}+\mu_{p}\gamma$$
  Herschel–Bulkely model:
  (2) $$\tau =\tau_{y}+K\gamma^{n}$$
  where:
τ is the shear stress, γ is the shear rate, τ_0_ is the yield point, μ_p_ is the plastic viscosity, τ_y_ is the yield point under this model, K is the flow consistency coefficient, and n is the fluid behavior index.

**Figure 8:**
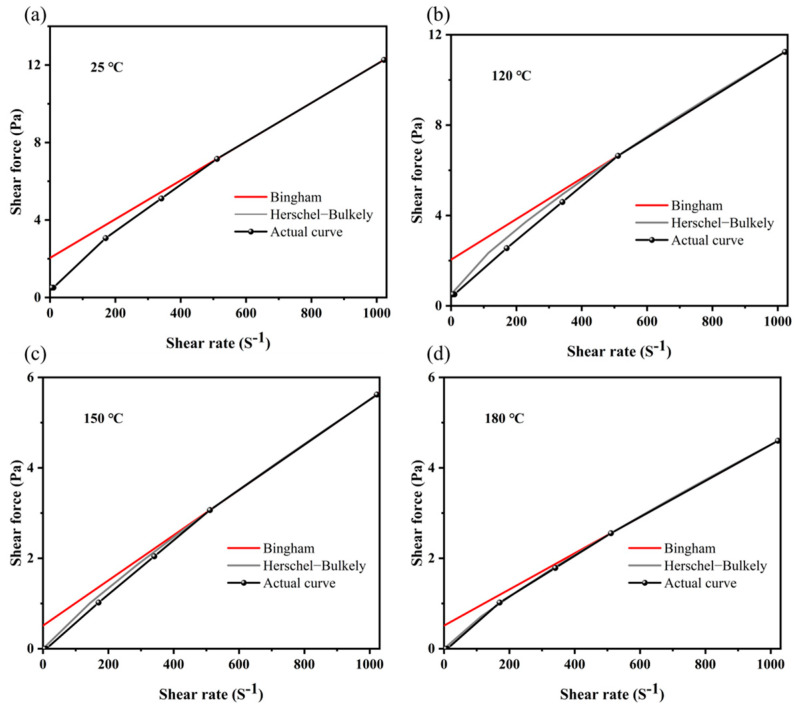
Rheological curve fitting for the drilling fluid gel system after aging at different temperatures: (**a**) 25 °C, (**b**) 120 °C, (**c**) 150 °C, and (**d**) 180 °C.

In general, a drilling fluid gel system is required to have a higher viscosity at a lower shear rate to carry or suspend the cuttings in the borehole, and a lower viscosity at a higher shear rate to pump quickly to the bottom of the borehole and release the cuttings easily [41]. First, the rheological properties of the drilling fluid gel system with the freshwater base slurry were studied at different aging temperatures. The fitting equations and curves of different rheological models are shown in Table 2 and Figure 8. It was found that the rheological curve of the drilling fluid gel system was more consistent with the Herschel–Barclay model. The linear correlation coefficients R^2^ of the aging temperature at 25 °C, 120 °C, 150 °C, and 180 °C were 0.9986, 0.9949, 0.9980, and 0.9992, respectively.

In addition, the effects of MW−HPAN on the rheological parameters of the drilling fluid gel system at different aging temperatures, such as AV, PV, YP, and RYP, were investigated. Temperature has a very important influence on the stability of a drilling fluid gel system, which is reflected in the interaction between clay particles and the polymer additive. First, the clay particles will disperse, agglomerate, and passivate at high temperatures [42,43]. Second, a high temperature will lead to degradation, a change in the side chain functional groups, or high-temperature cross-linking of the polymer [44]. Furthermore, the high-temperature effect will weaken the adsorption capacity of the polymer treatment agent on the clay surface, which is called high-temperature desorption [45]. At the same time, a high temperature will also reduce the hydration ability of the treatment agent on the clay surface, reduce the thickness of the hydration film, and weaken the adhesion protection performance, which is called high-temperature dehydration [46,47].

**Figure 9:**
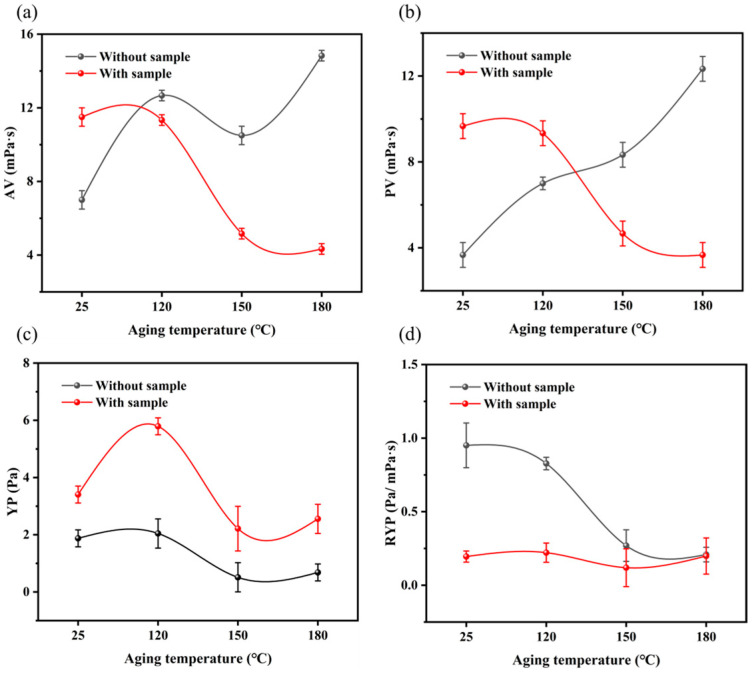
The influence of samples on rheological parameters of drilling fluid gel system after aging at different temperatures: (**a**) apparent viscosity, (**b**) plastic viscosity, (**c**) yield point, and (**d**) the ratio of yield point to plastic viscosity.

As shown in Figure 9, the AV and PV of the drilling fluid gel system increased the overall after aging at different temperatures and decreased slightly at 150 °C without the sample. The YP first increased to a maximum value at 120 °C, gradually decreased and reached a minimum value at 150 °C, and then increased with temperature. The RYP decreased overall. These phenomena were related to the dynamic equilibrium of high-temperature dispersion, agglomeration, and passivation of clay particles [48].

The AV of the drilling fluid gel system at room temperature was increased with the sample, which was because the MW−HPAN molecules in the drilling fluid gel system were in an irregular nematic conformation, the friction within the macromolecule was greater, and, at the same time, it adsorbed water molecules and increased the internal friction between polymers and water molecules. In addition, the cyano and amide functional groups of the MW−HPAN molecular side chain were adsorbed on the clay surface by hydrogen bonding and formed a spatial network structure. Under the combined effect of these factors, the AV, PV, YP, and RYP of the drilling fluid gel system increased. As the aging temperature increased, the AV and PV of the drilling fluid gel system gradually decreased. This was because the high temperature broke the MW−HPAN molecular chain. In addition, the cyano and amide functional groups underwent hydrolysis at high temperatures, even leading to the breakage of the functional groups and the main chain [49]. The adsorption and hydration capacity of MW−HPAN on clay surfaces weakened with the temperature, resulting in a reduction in the thickness of the hydration film on clay particles. These two effects negatively impact the adhesive protection ability of MW−HPAN [44,50]. The RYP of the drilling fluid gel system remained within a certain range with increasing aging temperature, indicating a certain level of high-temperature resistance of MW−HPAN. It was found that the drilling fluid gel system with MW−HPAN had good rheological properties.

#### 2.3.2. Filtration Performance of the Drilling Fluid Gel System

In the drilling process, the key to controlling the filtration performance of the drilling fluid gel system is to improve the quality of the mud cake, which involves an enhancement in the compactness, shear resistance, and lubricity of the mud cake [51]. Bentonite, a commonly used slurry material for water-based drilling fluid gel systems, is beneficial for forming dense mud cakes that prevent water from penetrating into the formation. However, the instability and aggregation of bentonite can occur in high temperature and high salt content environments, leading to increased porosity and permeability of the filter cake and an increasing filtration loss [52].

**Figure 10:**
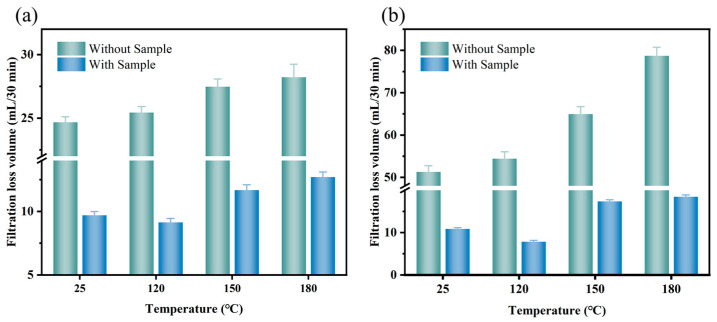
The filtration loss of the gel after aging at different temperatures. (**a**) Freshwater base slurry: the sample dosage was 0.3%; and (**b**) 4%NaCl brine base slurry: the sample dosage was 1.2%.

The filtration performance of the drilling fluid gel system with MW−HPAN was measured under different aging temperatures and salt concentrations. First, the filtration performance of freshwater base slurry before and after aging at 120 °C, 150 °C, and 180 °C was evaluated, and the sample dosage was 0.3%. As shown in Figure 10a, the filtration loss of the freshwater base slurry without samples before aging is 24.9 mL/30 min, while after aging at 120 °C, 150 °C and 180 °C, the filtration loss increases to 25.2 mL, 27.1 mL, and 28.3 mL, respectively. The increase in filtration loss means that aging will affect the filtration performance of the drilling fluid gel system. However, the final filter loss with samples before and after aging is significantly reduced. When the sample addition is 0.3%, the filtration loss before and after aging at 120 °C, 150 °C, and 180 °C is reduced to 9.6 mL, 8.8 mL, 11.6 mL, and 12.2 mL, respectively, which meets the API recommended requirements. It can be observed that filter loss increases slightly with the temperature, which means the increasing aging temperature does not affect the filtration reduction performance of the sample.

Second, in order to evaluate the salt resistance of the sample, the filtration performance of the 4% NaCl brine base slurry before and after aging at 25 °C, 120 °C, 150 °C and 180 °C was tested, and the sample dosage was 1.2%. As shown in Figure 10b, the filtration loss volume of 4% NaCl brine base slurry before aging was 51.0 mL, and it increased continuously with the increase in aging temperature. The filtration loss increased to 75.6 mL after aging at 180 °C, which was much higher than that of the freshwater base slurry, indicating that the filtration performance of the drilling fluid gel system became worse after the synergistic damage effect of high temperature and inorganic salt pollution. However, after adding 1.2% of the sample as a filtrate reducer to the 4% NaCl brine base slurry, the drilling fluid gel system can maintain a low filtration loss before and after hot rolling. After aging at 180 °C, the filtration loss is 17.8 mL, and the viscosity of the drilling fluid gel system is not high. It can be seen that viscosity may not be the main factor affecting the filtration performance of the drilling fluid gel system after aging. There are many active functional groups on the MW−HPAN molecular chain, of which the adsorption groups (cyano and amide groups) can be firmly adsorbed on the surface of clay particles by the formation of hydrogen bonds with the oxygen atoms on bentonite, and the presence of carboxyl groups improves the salt resistance of sample [53]. Furthermore, the rigidity of the C-C and cyano group in the MW−HPAN molecule enhances its high-temperature resistance. These factors are responsible for the good temperature and salt resistance performance of MW−HPAN.

#### 2.3.3. Microstructural Analysis of the Drilling Fluid Gel System

The DLVO theory is the classical colloidal stability theory that explains the stability of charged colloidal particles in the liquid phase, showing that the stability of colloidal particles is determined by the combined effect of van der Waals’ gravitational and electrostatic repulsive forces between the particles. The diffusion double-layer of colloidal particles is divided into an adsorption layer, formed by the attraction between the colloidal particles and the hetero ions, and a diffusion layer, formed by the thermal movement of the hetero ions. According to the DLVO colloidal stability theory, high temperature and cationic contamination will compress the diffuse double layer of negatively charged clay in a drilling fluid gel system, destroy the rheological and filtration properties and reducing the stability of the drilling fluid gel system, which easily produce a thick and loose filter cake that leads to an increase in filtration loss. Filtrate reducer can prevent the negative effect by forming thin and dense filter cake, and thus achieve the effect of reducing filtration loss [52,54]. 

**Figure 11:**
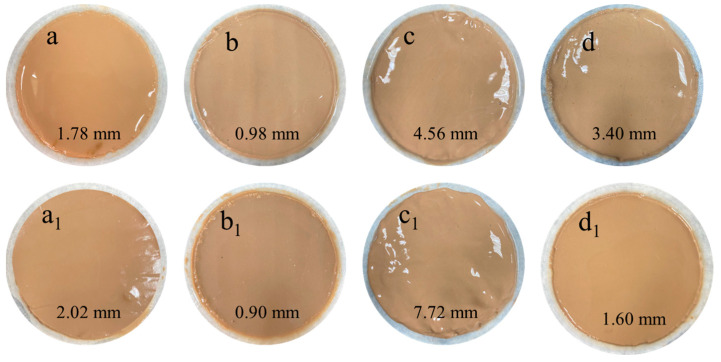
Images of the filter cake with and without the sample after aging (at 180 °C for 16 h) and before aging: (**a**) without the sample before aging (freshwater base slurry), (**b**) with the sample before aging (freshwater base slurry), (**c**) without the sample before aging (4% NaCl base slurry), (**d**) with the sample before aging (4% NaCl base slurry), (**a_1_**) without the sample after aging (freshwater base slurry), (**b_1_**) with the sample after aging (freshwater base slurry), (**c_1_**) without the sample after aging (4% NaCl base slurry), and (**d_1_**) with the sample after aging (4% NaCl base slurry).

The thickness of the fresh filter cake was measured, and the microstructure of the filter cake after freeze-drying was observed using SEM to evaluate the effect of MW−HPAN on the quality of the filter cake. The thickness of the filter cake from the freshwater base slurry without the sample before aging was 1.78 mm (Figure 11a_1_); under the joint destruction of cation and high temperature, the structure of the filter cake became loose, and the thickness of the filter cake from the 4% NaCl brine base slurry was 7.72 mm (Figure 11c_1_), indicating that the stability of the suspension system decreased and the filter loss increased. However, the thickness of the filter cake with the sample was significantly reduced before and after aging. The thickness of the filter cake of the 4% NaCl brine base slurry was reduced from 7.72 mm to 1.60 mm (Figure 11d_1_), indicating that MW−HPAN could hinder the destruction effect of the electrolyte ion and high temperature on the filter cake.

**Figure 12:**
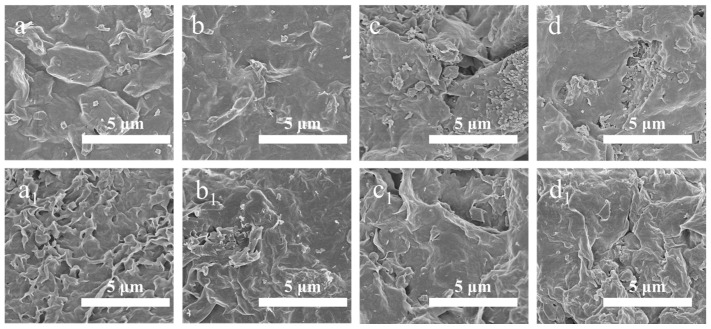
SEM images of the filter cake with and without the sample after aging (at 180 °C for 16 h) and before aging: (**a**) without the sample before aging (freshwater base slurry), (**b**) with the sample before aging (freshwater base slurry), (**c**) without the sample before aging (4% NaCl base slurry), (**d**) with the sample before aging (4% NaCl base slurry), (**a_1_**) without the sample after aging (freshwater base slurry), (**b_1_**) with the sample after aging (freshwater base slurry), (**c_1_**) without the sample after aging (4% NaCl base slurry), and (**d_1_**) with the sample after aging (4% NaCl base slurry). (×10.0 k magnification).

The SEM images were consistent with the above findings (Figure 12). The agglomeration of clay appeared on the surface of the filter cake obtained from the base slurry, and a large number of wrinkles, cracks, and pores were formed after aging (Figure 12a_1_). These phenomena were more significant with the effect of cations (Figure 12c_1_). The increase in the permeability of the filter cake led to a rapid increase in filter loss. However, the surface of the filter cake after adding the sample to the base slurry was smooth and dense, and it looked like the polymer film covered it (Figure 12b_1_). Under the destructive effect of high temperature and cation, MW−HPAN could reduce the agglomeration of clay, prevent the formation of wrinkles and cracks, reduce the permeability of filter cake, and obviously improve the filtration performance of the drilling fluid gel system. The law of variation in filter cake quality was consistent with the variation in filter loss in Figure 10.

#### 2.3.4. Particle Size Analysis of the Drilling Fluid Gel System

A reasonable size distribution of clay particles in a drilling fluid gel system is required to form a low-permeability mud cake. Larger clay particles accumulate with each other to form a relatively strong bridging structure, while small-sized clay particles enter into the cracks and pores, acting as a sealer in the structure of the filter cake, which is conducive to the formation of a stable and dense filter cake and reduces the penetration of water [55,56].

**Figure 13:**
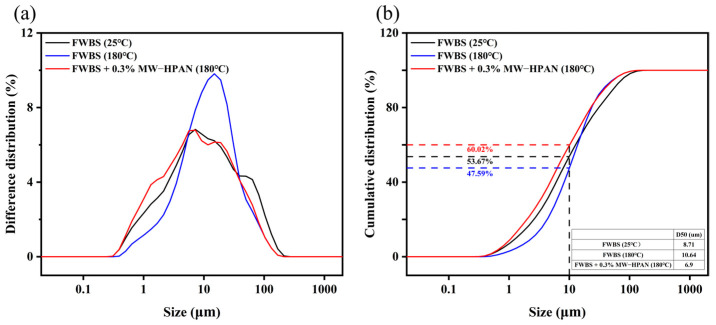
Particle size distribution of freshwater base slurry (FWBS) before and after aging. (**a**) The difference distribution and (**b**) the cumulative distribution.

As shown in Figure 13, the particle size distribution of the freshwater base slurry without the sample before aging was relatively reasonable: about 53.67% of the clay particles were smaller than 10 μm, and the average particle size of the system was about 8.71 μm. The particle size distribution of the freshwater base slurry was narrower after aging at 180 °C, and the average particle size of the system increased to 10.64 μm, indicating the aggregation of clay particles at high temperatures. However, the drilling fluid gel system still maintained a suitable particle size distribution after aging with the addition of MW−HPAN. Specifically, 60.02% of the particle sizes in the system were less than 10 μm, and the average particle size was reduced to 6.90 μm, which was due to the firm adsorption between the sample and the clay surface through hydrogen bonding, preventing the clay particles from flocculating and becoming larger. In addition, MW−HPAN stabilized the fine particles that were not bound into large particles by adsorption, which increased the proportion of fine particles and was conducive to the formation of a dense filter cake, called the protective effect of colloid. The experimental results showed that the high temperature could hardly destroy the colloid stability (colloid stability refers to the invariability in the viscosity, particle size, and other properties of the colloidal solution system to a certain extent) of the drilling fluid gel system with MW−HPAN, and the results were consistent with the observations of the filter cake (Figure 11 and Figure 12).

### 2.4. Comparison with Commercially Available Products

**Figure 14:**
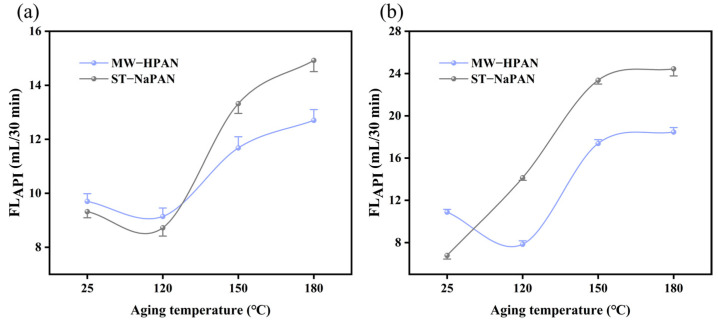
Filtration performance of MW–HPAN and ST–NaPAN in drilling fluid gel systems. (**a**) The freshwater base slurry and (**b**) the 4% NaCl brine base slurry.

The obtained sample, MW−HPAN, is essentially a hydrolyzed polyacrylonitrile sodium salt, and we denoted it as MW−HPAN since it was prepared using a novel micro-water method. Its properties were compared with hydrolyzed polyacrylonitrile sodium salt products synthesized with the conventional process. The filtration loss performance of MW−HPAN and commercially available hydrolyzed polyacrylonitrile sodium salt (ST–NaPAN was selected as a representative product for comparison) in the freshwater- and 4% brine-based slurries before and after aging at different temperatures was evaluated. As shown in Figure 14a, the filtration loss of ST–NaPAN was slightly lower than that of MW–HPAN when the aging temperature was lower than 120 °C in the freshwater base slurry, but the filtration loss reduction effect of MW−HPAN was obviously better than ST–NaPAN when the aging temperature was higher than 120 °C. This variation trend was more significant in the brine base slurry (Figure 14b): the filtration loss volume of MW–HPAN was 10.8 mL before aging, which was higher than ST–NaPAN, but it still met the requirements of API recommendations. The filtration loss reduction performance of ST–NaPAN decreased drastically even after aging at a lower temperature (120 °C). However, the filtration loss value of MW–HPAN first decreased and then increased with an increase in the aging temperature, and the filtration loss reduction performance could still maintain a better level after aging at 180 °C.

These results indicate that MW–HPAN has a significantly better anti-temperature and anti-salt performance than ST–NaPAN as a filtrate reducer for drilling fluid gel systems. This is due to the difference between the two hydrolysis reaction processes. First, sufficient water is required in the traditional hydrolysis process as a solvent. Due to the agglomeration of acrylic fiber and the heterogeneity of the reaction, the hydrolysis process takes a long time, during which PAN undergoes more severe degradation in this highly alkaline environment. Second, the drying time is long due to the high water retention of the semi-finished product, during which further hydrolysis will occur. Therefore, a low molecular weight and high hydrolysis degree of the hydrolyzed polyacrylonitrile product (HPAN) will be obtained, and the reduction in molecular weight will greatly reduce the temperature and salt resistance of HPAN. Furthermore, the stability of the performance of the samples will vary from batch to batch due to the difference in the purity of the waste fibers. In this study, the hydrolysis of polyacrylonitrile with hydroplastization followed by the micro-water hydrolysis method could solve the above problems. Hydroplastization can separate and purify low-purity waste polyacrylonitrile fibers into a high-purity polyacrylonitrile powder, and the HPAN with a moderate hydrolysis degree can be obtained in a short time using the micro-water “vapor-solid” reaction at high temperature. There is no degradation of molecular weight in this novel process, and the drying cost is low. The performance of the HPAN can be significantly improved with this new hydrolysis process.

### 2.5. Mechanism of Filtration Loss Reduction

**Figure 15:**
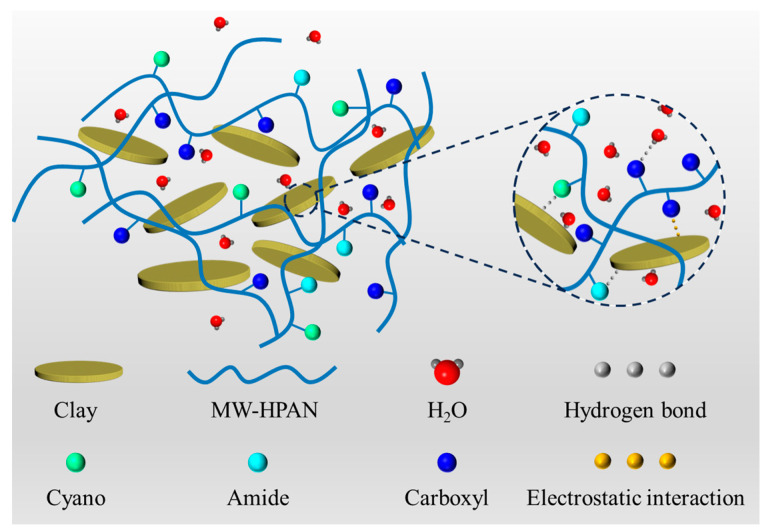
Schematic diagram of the filtration reduction mechanism of MW–HPAN [57].

Clay particles can form stable ionic and hydrogen bonds with the hydration group and adsorption group of the polymer, respectively. These two effects contribute to the polymer being firmly adsorbed on clay particles and the formation of a stable spatial lattice structure, which is conducive to the formation of a dense mud cake and reducing the filtration loss volume [57]. As shown in Figure 15, the filter loss-reducing agent MW−HPAN obtained with the micro-water hydrolysis method contains carboxyl, amide, and unhydrolyzed cyano groups on the molecular chain, in which the adsorption groups (cyano and amide) can form a hydrogen bond with the oxygen atoms on bentonite, so that the MW–HPAN is firmly adsorbed on the surface of the clay, generating a good colloid protective effect on the suspension. A reasonable particle distribution of the drilling fluid gel system after aging at high temperatures can be obtained with MW−HPAN. A large number of small particles are more conducive to filling the cracks and pores, forming a dense filter cake and reducing the permeability of the filter cake. Furthermore, the molecular chains of MW–HPAN can be stretched in the drilling fluid gel system with the presence of a hydration group (carboxyl group) and adsorb free water into bound water, forming a hydration layer around the molecular chain. This large and thick adsorption of the hydration film can still be effective in preventing the flocculation of clay particles even after aging at high temperatures, achieving the effect of reducing filtration loss.

## 3. Conclusions

The experimental results indicate that the reaction of hydrolysis of polyacrylonitrile using the micro-water method was successful. MW–HPAN obtained with the reaction at m_PAN_:m_NaOH_:m_H2O_ = 1:0.5:0.6 at 160 °C for 1 h can be used as a filtrate reducer for drilling fluid gel systems with excellent performance. The hydrolysis of polyacrylonitrile is a process in which hydrophobic cyano groups react under alkaline conditions to form hydrophilic amide and carboxyl groups. The addition of a small number of water molecules makes the microenvironment of the hydrolysis reaction system different from that of the liquid phase reaction, which results in a localized high concentration at the reaction site and greatly improves the efficiency of the reaction. The results of the elemental analysis showed that its hydrolysis degree could reach 73.21% in a short time, which was unattainable in traditional processes under the same conditions. MW–HPAN showed better temperature and salt resistance properties than commercially available HPAN products in the water-based drilling fluid gel system. The method of preparing MW–HPAN using micro-water hydrolysis has obvious economic benefits and a broad market prospect.

## 4. Materials and Methods

### 4.1. Materials

Waste acrylic fibers (industrial grade) used in the experiment were purchased from a textile factory in Luancheng City (Shijiazhuang, China), which consisted of polyacrylonitrile and other blended fibers (denoted as LC–PAN). Sodium hydroxide, sulphonic acid, and anhydrous sodium carbonate, used without any further purification, were analytical grade and purchased from Sinopharm Chemical Reagents Co., Ltd. (Beijing, China). Industrial grade hydrolyzed polyacrylonitrile sodium salt (Na-HPAN, Na-HPAN is an abbreviation for the product of alkaline hydrolysis of polyacrylonitrile) was obtained from Baoding Santuo Chemical Products Co., Ltd. (Baoding, China). Calcium bentonite for the base slurry was purchased from Drilling & Exploration Engineering Co., Ltd. (Tianjin, China). The England evaluation clay for the drilling fluid gel system test was purchased from the Beijing Institute of Exploration Engineering (Beijing, China).

### 4.2. Synthesis of Na-HPAN with the Micro-Water Method

#### 4.2.1. Hydroplastization of Waste Acrylic Fibers

**Figure 16:**
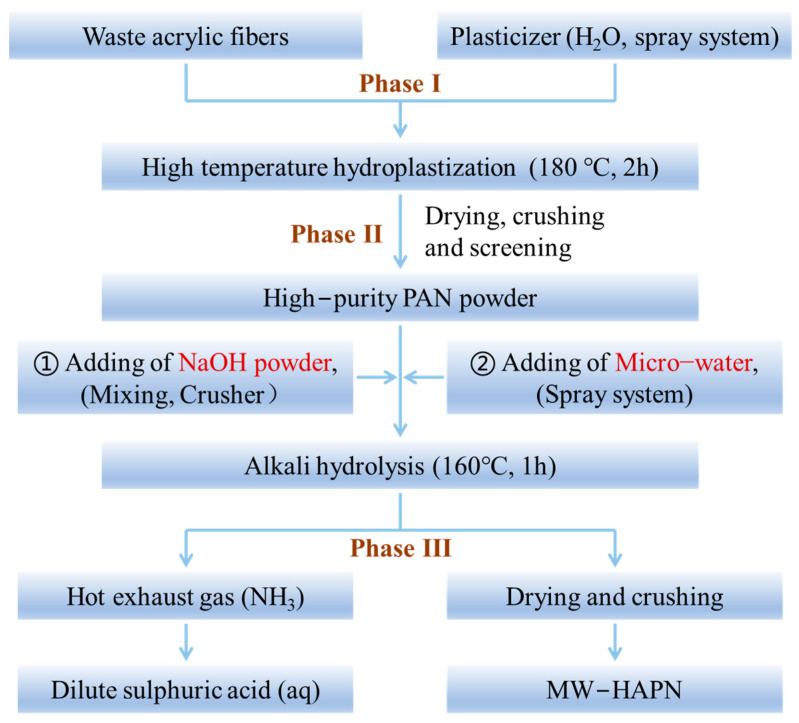
Schematic diagram of polyacrylonitrile hydrolysis using the micro-water method.

After opening, the waste acrylic fibers (40 g) and water (40 g) were added to a digestion tank and then placed in a hot-roller furnace. The hydroplastization reaction was carried out at 180 °C for 2 h. During the reaction, the structure of the acrylic fibers was destroyed, while the structure of the non-acrylic blended fibers was unchanged. The dry plasticized acrylic fibers were crushed with a grinder and separated from the other blended fibers with a 60-mesh screen to obtain a high-purity polyacrylonitrile powder (Figure 16, Phase I).

#### 4.2.2. Alkaline Hydrolysis of the Polyacrylonitrile Powder with the Micro-Water Method

The obtained polyacrylonitrile powder (20 g) and sodium hydroxide (10 g) were mixed firmly in a grinder and poured into a reaction kettle with an outlet valve stem, to which water (12 g) was added in two batches at the same time (Figure 16, Phase II). After sealing, the kettle was placed in the hot-roller furnace for hydrolysis at 160 °C for 1 h, and then the kettle was removed. The ammonia gas and water vapor in the kettle were immediately absorbed with a dilute sulphonic acid solution through the valve stem. The sample was dried with residual heat, and the hydrolyzed polyacrylonitrile sodium salt was obtained using the micro-water method (MW–HPAN) after crushing (Figure 16, Phase III). The product was washed three times alternately with water and anhydrous ethanol and dried at 70 °C for 24 h. The dried product was ground to powder for subsequent characterization.

### 4.3. Structure Characterization Techniques

#### 4.3.1. Elemental Analysis (EA)

The organic elemental content of the samples before and after hydrolysis was determined using a UNICUBE organic elemental analyzer (Elementar Trading Co., Ltd.; Shanghai, China). The limit of detection (LOD) was 50 ppm with an accuracy of 0.01%.

#### 4.3.2. X-ray Diffraction (XRD)

The crystal structures of the samples were analyzed using a Bruker D8 Advance X-ray diffractometer (Hongkong, China) equipped with a Cu-Kα radiation source (λ = 0.154 nm), and the diffraction patterns from 0° to 60° were collected at a scan rate of 2°/min.

#### 4.3.3. Scanning Electron Microscope (SEM)

The dry samples were mounted on an aluminum holder and an ion sputtering device (Model E-1010, Hitachi; Tokyo, Japan) was used for gold sputter coating to make it conductive. Then, the morphologies of the samples were characterized using a Hitachi SU8020 scanning electron microscope (Tokyo, Japan) operating at an accelerating voltage of 3 kV.

#### 4.3.4. Fourier Transform Infrared Spectra (FT-IR)

The sample testing tablets were carried out using the KBr compression method and characterized with a Nicolet IS10 FT-IR spectrometer (Madison, WI, USA) in the range of 4000 cm^−1^ to 500 cm^−1^ with the resolution of 4 cm^−1^ and the signal-noise ratio was 50,000:1. All spectrums were obtained by accumulating 64 scans.

#### 4.3.5. Thermogravimetric (TG)

The thermal decomposition behaviors of the samples were investigated using a Netzsch STA449F3 thermogravimetric analyzer (Selb, Germany) under a nitrogen atmosphere and a nitrogen flow rate of 50 mL/min. The samples were placed in a clean crucible and heated from 30 °C to 600 °C at a heating rate of 10 °C/min. The derivative thermogravimetric analysis (DTG) curves were obtained by applying the first-order derivative to the temperature with the TGA data.

#### 4.3.6. Particle Size Analysis

The particle size distribution of the sample was measured using a Mastersizer 2000 laser particle sizer (Malvern Instruments Co., Ltd., Shanghai, China). Before each test, the samples were ultrasonically dispersed in ethanol solution for 5 min, and three parallel experimental tests were carried out. The shading rate was set at 14%, and the measuring range of the instrument was 0.02–2000 μm.

### 4.4. Comprehensive Performance Evaluation of the Water-Based Drilling Fluid Gel System

**Figure 17:**
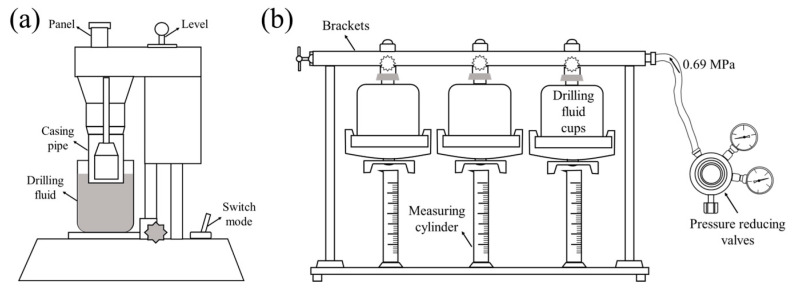
Schematic diagram of drilling fluid gel system performance testing instruments. (**a**) Six-speed rotational viscometer and (**b**) medium-pressure filter loss meter.

The performance of water-based drilling fluid gel systems, including filtration properties, temperature, salt resistance, and rheological properties were evaluated according to the American Petroleum Institute Recommended Practices (API RP 13B-1-2019). All performance evaluation experiments were carried out at least three times, and the mean and standard deviation of the parallel experiments were calculated. The apparatus used for the test is shown in Figure 17.

#### 4.4.1. Preparation of the Freshwater Base Slurry

In total, 0.525 g of anhydrous sodium carbonate and 15.0 g of bentonite were added to a high stirring cup containing 350 mL of deionized water and stirred at high speed for 20 min, with at least two stops during this time to scrape off clay adhering to the wall of the cup. The mixture was then cured at room temperature for 24 h as a freshwater base slurry.

#### 4.4.2. Preparation of the 4% NaCl Brine Base Slurry

In total, 4 g of sodium chloride, 2.1 g of anhydrous sodium carbonate, 60.0 g of drilling fluid gel system slurry with bentonite were added to a high stirring cup containing 350 mL of deionized water and stirred at high speed for 20 min, with at least two pauses to scrape off the clay attached to the wall of the cup. Then, the above dispersion was cured at room temperature for 24 h as a 4% NaCl brine base slurry.

#### 4.4.3. Measurement of API Filtration Performance

The API filtration loss of the prepared drilling fluid gel systems was determined using a medium-pressure filter loss meter (SD3, Qingdao Tongchun Petroleum Instrument Co., Ltd., Qingdao, China) and standard Fann filter paper. A certain amount of the sample was added to the well-cured 350 mL base slurry (freshwater or 4% brine) and stirred at high speed for 20 min. Then, the mixture was poured into an airtight container and conditioned at room temperature. After 16 h, the conditioned drilling fluid gel systems were stirred at high speed for 5 min and poured into a filter loss cup until the level of the fluid was tangent to the graduated line. A pressure of 100 psi (0.69 MPa) was applied with a nitrogen cylinder. The 30-minute filtrate volume was collected to indicate the API filtration loss performance of the drilling fluid gel system. In addition, the filter cake was carefully removed from the loss of the filtration cup and slowly washed off the flowing mud on its surface with water, and its thickness was then determined using a vernier caliper.

#### 4.4.4. Determination of Rheological Properties of Drilling Fluid Gel Systems

A certain amount of the sample was added to the well-conditioned 350 mL base slurry (freshwater or 4% brine) and stirred at high speed for 20 min before being conditioned at room temperature for 16 h. After 16 h, the sample was stirred at high speed for 5 min and then poured into the drilling fluid gel system beaker of a Fanns model 351 six-speed rotational viscometer (ZNN-D6, Qingdao Haitongda Special Instrument Co., Ltd., Qingdao, China) until the surface of the fluid was tangent to the scale of the viscometer, and the stable readings at different rotational speeds (600 rpm, 300 rpm, 200 rpm, 100 rpm, 6 rpm, 3 rpm) were then recorded. The apparent viscosity (AV), plastic viscosity (PV), yield point (YP), and the ratio of yield point to plastic viscosity (RYP) of the drilling fluid gel system were calculated using the following equations.

(3) $$\mathrm{AV}=\frac{1}{2}\Phi 600$$

(4) PV=Φ600−Φ300

(5) $$YP=\frac{1}{2}\Phi 300-PV$$

(6) RYP=YP/PV

where:

AV is the apparent viscosity (mPa·s); PV is the plastic viscosity (mPa·s); YP is the yield point (Pa); RYP is the ratio of the yield point to the plastic viscosity; Φ600 is the dial reading of the 6-speed rotational viscometer at 600 r/min (dia); and Φ300 is the dial reading of the 6-speed rotational viscometer at 300 r/min (dia);

#### 4.4.5. Measurement of the Temperature Resistance of Drilling Fluid Gel Systems

A certain amount of the sample was added to the well-maintained 350 mL base slurry (freshwater or 4% brine) and stirred at high speed for 20 min. Then, the mixture was poured into a high-temperature aging tank, sealed, and placed in a high-temperature aging oven (XGRL-5, Qingdao Haitongda Special Instrument Co., Ltd., Qingdao, China), and aging was carried out at different temperatures for 16 h. After 16 h, the drilling fluid gel systems were allowed to cool to room temperature, high-speed stirring was carried out for 5 min, and then the filtration loss properties and rheological properties were determined.

#### 4.4.6. Particle Size Analysis of Drilling Fluid Gel Systems

The particle size distribution of bentonite particles in the drilling fluid gel system before and after aging was measured using a Bettersize 2000 laser particle sizer (Dandong Bettersize Instruments Co., Ltd., Dandong, China). Water was chosen as the dispersion medium, sodium hexametaphosphate was used as the dispersant, the shading rate was set at 12%, and the measuring range of the instrument was 0.02–2000 μm.

#### 4.4.7. Morphologies of Drilling Fluid Gel System Mud Cakes

The mud cake obtained from the API filtration loss test was immersed in liquid nitrogen and frozen for 2–3 min. Then, it was removed and rapidly transferred to a vacuum freeze dryer (ZLGJ-18, Zhengzhou Huachen Instrument Co., Ltd., Zhengzhou, China) and vacuum lyophilized at −30 °C for 48 h. The surface of the mud cake was then gold plated, and the morphology was characterized using a Hitachi SU8020 scanning electron microscope at an accelerating voltage of 3 kV.

## Figures and Tables

**Table 1:** Elemental content of LC−PAN and MW−HPAN.

Sample	Elemental Content (wt%)			C/N	C/O
	C	N	O		
LC−PAN	61.60	21.24	9.31	2.90	6.62
MW−HPAN	36.89	3.41	42.66	10.82	0.86

**Table 2:** Rheological fitting equation of the drilling fluid at different aging temperatures.

Temperature °C	Flow Pattern			
	Bingham		Herschel–Bulkely	
	Equation	R^2^	Equation	R^2^
25	τ = 2.044 + 0.0100 × γ	0.9430	τ = 0.511 + 0.0392 × γ^0.8231^	0.9986
120	τ = 2.044 + 0.0090 × γ	0.9247	τ = 0.511 + 0.0399 × γ^0.8074^	0.9949
150	τ = 0.511 + 0.0050 × γ	0.9677	τ = 0 + 0.0131 × γ^0.8745^	0.9980
180	τ = 0.511 + 0.0040 × γ	0.9595	τ = 0 + 0.0129 × γ^0.8480^	0.9992

## Data Availability

All data and materials are available on request from the corresponding author. The data are not publicly available due to ongoing researches using a part of the data.
